# Editorial: Safety and efficacy of CRISPR/Cas-based genome editing tools: applications and considerations in cell and gene therapy

**DOI:** 10.3389/fimmu.2026.1809809

**Published:** 2026-03-06

**Authors:** Karl Petri, Samuele Ferrari, Beatrice Claudia Cianciotti

**Affiliations:** 1 Chair of Cellular Immunotherapy, Medical Clinic and Policlinic II, University Hospital Würzburg, Würzburg, Germany; 2 National Center for Tumor Diseases (NCT), Site WERA, Würzburg, Germany; 3 Bavarian Center for Cancer Research (BZKF), Lighthouse Cellular Immunotherapies, Würzburg, Germany; 4 San Raffaele Telethon Institute for Gene Therapy, IRCCS San Raffaele Scientific Institute, Milan, Italy; 5 Vita-Salute San Raffaele University, Milan, Italy; 6 Mucosal Immunology and Microbiota Lab, IRCCS Humanitas Research Hospital, Milan, Italy

**Keywords:** CRISPR/Cas, gene therapeutic target, genome editing, immunotherapy, safety

CRISPR/Cas−based immune cell engineering is undergoing a rapid transition from assessing experimental feasibility to the production of clinical−grade therapeutics. Since the first CRISPR-based therapy received regulatory approval in late 2023, the focus of the scientific community has increasingly shifted from the proof-of-concept demonstration of genetic modification to the improvement of safety and efficacy profiles required for clinical applications. The Research Topic is designed to bridge mechanistic immunology, genome-editing technology, and translational safety requirements around CRISPR/Cas tools in cell and gene therapy. The four contributions cover this spectrum by (i) reviewing CRISPR editing of primary T and NK cells for cancer immunotherapy, (ii) detailing knock−in strategies in human B cells and lymphoma, (iii) conceptualizing safety−assessment platforms for CRISPR−edited NK cells, and (iv) providing an early example of CRISPR−mediated modulation of T−cell signaling.

In the comprehensive review, Azangou-Khyavy et al. delineate the CRISPR/Cas9-based dual-action strategy in oncology: the direct disruption of tumor cell survival pathways and the immune cell engineering to overcome the immunosuppressive tumor microenvironment. Their review highlights the potential of disrupting inhibitory receptors (such as PD-1) and the strategic advantage of site-specific CAR insertion into the *TRAC* locus to create universal T-cell products with reduced exhaustion and alloreactivity.

The practical application of these concepts is exemplified by the original research article by Bray et al. The protein tyrosine phosphatase PTPN22 is a critical negative regulator of T-cell receptor (TCR) signaling, and its variants have been linked to numerous autoimmune diseases. By exploiting CRISPR/Cas9 to generate the first human PTPN22-knockout T-cell lines, the authors were able to isolate the functional effects of this gene. Their findings demonstrate that the loss of PTPN22 enhances the expression of the activation marker CD69 and the production of IL-2, particularly in response to weak-antigen stimulation. This research not only clarifies the mechanism of a key regulator in autoimmune pathogenesis but also suggests potential strategies for engineering T cells with lower activation thresholds for immunotherapy.

While T-cell therapies remain the gold standard, the field is expanding toward other immune subsets. The perspective by Lund et al. shifts the focus from knock−out to knock−in genome editing strategies. Their work provides robust protocols for modeling the genetic heterogeneity of B-cell malignancies like diffuse large B-cell lymphoma (DLBCL). By enabling the introduction of specific oncogenic mutations into primary B cells, this methodology facilitates more accurate preclinical disease models and identifies potential targets for therapeutic intervention. This work provides a roadmap for using CRISPR to unlock the biology of hematological malignancies or generate preclinical models for regulatory-grade data.

Finally, as these therapies move toward the clinic, the safety address of the topic becomes the primary gatekeeper. Fazeli et al. argue that safety assessment must be an integral design principle rather than a final checklist. Focusing on Natural Killer (NK) cells (the emerging candidates for allogeneic therapy) the authors emphasize that technical enhancements, such as the expression of IL-15 to increase persistence, carry inherent risks of unintended genomic instability. They provide a comprehensive roadmap of safety platforms, ranging from in silico prediction to advanced *in vivo* humanized mouse models and organ-on-chip systems, ensuring that the next generation of edited cells can be rigorously investigated for clinical use.

Across the four contributions, a coherent roadmap emerges for the safe and effective use of CRISPR/Cas editing in immuno−oncology. Foundational mechanistic work in modelling T−cell biology informs target selection; broad reviews of T−cell editing and tumor−directed CRISPR strategies delineate novel therapeutic opportunities; knock−in methodologies enable precise disease modeling and translational hypothesis testing; and cell-specific safety platforms show how comprehensive risk assessment can be embedded into clinical product development.

